# Development of a Simple Risk Model to Predict Mortality in Patients With Osteosarcoma of the Extremity

**DOI:** 10.3389/fmed.2022.852529

**Published:** 2022-05-23

**Authors:** Yu Chen, Chao Li, Xin Wang, Zhiyong Liu, Zhigang Ren

**Affiliations:** ^1^Gene Hospital of Henan Province, Precision Medicine Center, The First Affiliated Hospital of Zhengzhou University, Zhengzhou, China; ^2^Academy of Medical Science, Zhengzhou University, Zhengzhou, China; ^3^Department of Orthopaedic Surgery, Affiliated Cancer Hospital of Zhengzhou University, Zhengzhou, China; ^4^Department of Infectious Diseases, The First Affiliated Hospital of Zhengzhou University, Zhengzhou, China

**Keywords:** osteosarcoma, clinical data, outcome, prognostic factors, risk model

## Abstract

**Background:**

Osteosarcoma (OS) is the most prevalent primary malignant bone cancer with poor prognosis. The aim of this study was to explore the prognostic factors that influence survival, and build up and validate a simple risk model to predict mortality in OS patients.

**Materials and Methods:**

This was a single-center retrospective cohort study. A total of 153 patients with newly diagnosed OS were enrolled as the training group. We analyzed the clinical data and outcomes of the OS patients. Prognostic risk factors were identified and evaluated by a logistic regression model with Markov Chain Monte Carlo simulation. The risk score was constructed based on the training group and was further validated using each patient.

**Results:**

Among the 153 patients, the mean (standard deviation) age was 21.6 (14.2) years, and 62 (40.5%) patients were females. The rate of in-hospital mortality of patients was 41.2% (95% CI, 31.6–50.7%). The candidate prognostic factors were selected and evaluated in relation to patient age, sex, tumor site (lower/upper extremity), tumor volume, intramedullary length of lesion, serum levels of alkaline phosphatase (ALP) and primary metastasis. However, only tumor size and primary metastasis were identified as independent prognostic indicators for patients with osteosarcoma. The risk model had a C-statistic of 0.7308 with a predictive range of 21.05–68.42%. Based on the distribution of the risk score, 24.8, 49.7 and 25.5% of patients were stratified into the high-, average- and low-risk groups for in-hospital mortality, with corresponding probabilities of 0.684, 0.329, and 0.210, respectively.

**Conclusion:**

A simple risk model was developed and validated to predict the prognosis for patients with osteosarcoma of the extremity at primary diagnosis. The simple risk score system could be used to stratify patients into different risk groups of in-hospital mortality and may help clinicians judge the outcomes of prognosis and establish appropriate surveillance strategies.

## Introduction

Osteosarcoma (OS) is one of the most common primary malignant bone tumors in children and adolescents, accounting for approximately two-thirds of the bone cancers diagnosed in the second decade of life (1–3). The incidence of OS is approximately one to three cases annually per one million people worldwide (1–3). OS is characterized by the presence of an osteoid matrix or immature bone, mainly frequent sites in the metaphysis and diaphysis of long bones (femur, tibia) (2), and can be broadly classified into three histologic subtypes (intramedullary, surface, and extraskeletal) (4). The pathogenesis and etiology of OS are still unclear. In the past few decades, the 5-year survival of patients with localized OS has considerably improved to 78% with the development of neoadjuvant chemotherapy and surgical techniques. However, the 5-year survival rate drops to 25% in cases with metastasis at diagnosis or relapse (5). Moreover, another study showed that the 5-year survival rate showed no significant improvement in patients with localized disease and no improvement in metastatic patients over the past four decades (6). Thus, improving the survival of OS patients has proved challenging, although the therapy for osteosarcoma is on the precipice of advancement. The historical invariability of survival outcomes and the limited number of predictive risk factors known to be active in the development of this disease facilitate clinical trials designed to identify efficacious prognostic factors in patients with osteosarcoma.

Currently, some clinical studies have been reported regarding identifying the prognostic factors that influence survival in osteosarcoma, including patient age, sex, tumor site and size, histologic subtype, presence and location of metastases, histologic response to chemotherapy and type of surgery and surgical margins (7–14). However, there is no standardized system for evaluating the prognostic factors correlated with survival among these studies. Specifically, there were variations in the statistical methods and the study population. Consequently, the results are somewhat inconsistent and even contradictory in the published importance of some variables (such as patient age, tumor site, and tumor size) (8–10, 13). However, predictive models are important tools to provide estimates of patient outcome (15). Moreover, it is imperative to explore effective prognostic models to predict the mortality of patients with OS.

Herein, we mined the relevant clinical data of patients with osteosarcoma (OS) of the extremities spanning 3 years from 2013 to 2015. We subsequently identified prognostic risk factors related to poor outcomes and then developed and validated a risk model to stratify patients into different risk groups of in-hospital mortality and help clinicians provide patients with appropriate surveillance strategies.

## Materials and Methods

### Patients and Study Design

**Figure 1:**
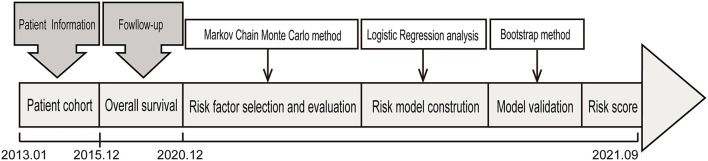
The design of the study. The patient cohort including 153 patients with newly diagnosed OS after a strict pathological diagnosis and exclusion process was used to identify prognostic factors for in-hospital mortality and was used for further evaluation and validation analysis.

Medical records of patients with OS of the extremities who were admitted to the Affiliated Cancer Hospital of Zhengzhou University between 1 January 2013 and 31 December 2015 with a minimum follow-up of 5 years were reviewed. The eligible patients for enrolment fulfilled the following criteria: typical radiographic and histologic features of primary, high-grade central osteosarcoma of the extremity. Exclusion criteria included non-extremity locations, low- or intermediate-grade osteosarcoma, treatment regimens that did not follow National Comprehensive Cancer Network (NCCN) guidelines, concomitant with previous history of cancer, and incomplete medical records. In total, we identified 153 patients who satisfied the prespecified study inclusion and exclusion criteria. The original 153 samples were used as the training group, and the bootstrap method was used as an internal test of the performance of the model (Figure 1). The ethics committee of the Affiliated Cancer Hospital of Zhengzhou University approved this study (No. 2017407).

### Potential Risk Factors and Outcome

The 7 candidate risk factors were easily collected, reliable, clinically important, and potentially associated with the outcome, including patient age, sex, tumor site (lower/upper limbs), tumor volume, intramedullary length of lesion, ALP and primary metastasis. The outcome was in-hospital mortality.

## Statistical Analysis

### Risk Factor Selection and Evaluation

Using the original sample with all candidate risk factors, we employ Markov Chain Monte Carlo (MCMC) simulation to select the primitive risk factors, which have a positive coefficient in more than 90% or <10% of the simulations and are regarded with a stable association with the outcome. The final risk model to predict the outcome was constructed by fitting a logistic regression model to the original sample using the selected risk factors by the MCMC simulation.

Due to the small sample size, the bootstrap method (16) was used to test model performance, and a total of 1,000 bootstrap samples were drawn with replacement of the same sample size as the original sample. Models were developed in the bootstrap samples and tested in the original sample.

**Figure 2:**
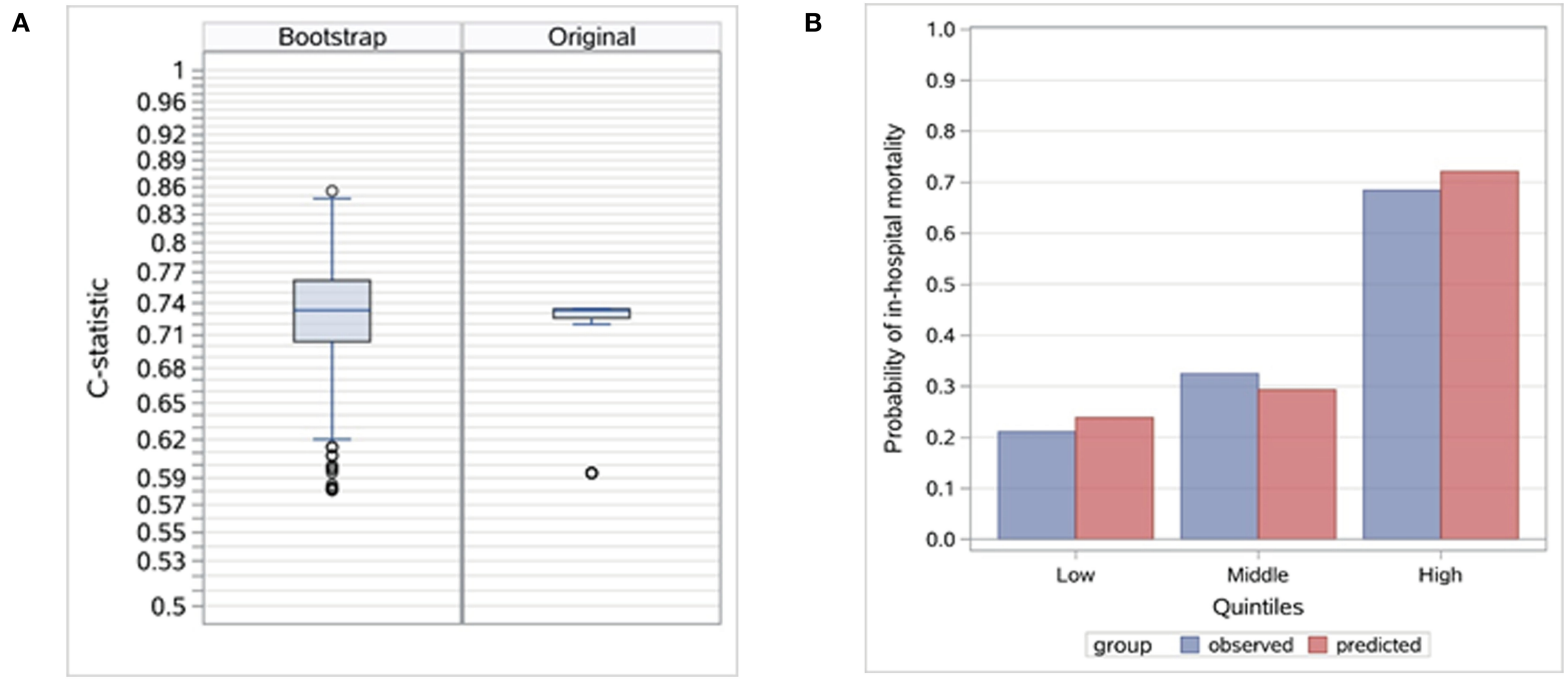
Evaluation of the risk model. **(A)** Distribution of c-statistic in bootstrap and original samples. Internally validated c-statistic is 0.7308. **(B)** Distribution of observed in-hospital mortality by stratified predictive probability.

We calculated the four indicators to evaluate the risk model performance. Discrimination was assessed with an internally validated c-statistic, and the distribution of the c-statistic for the bootstrap samples and the original sample (Figure 2A) are also presented. The internally validated calibration slope was used to measure calibration, and the observed in-hospital mortality in strata defined by quantiles of the predictive probabilities is presented (Figure 2B). We divided patients in the original sample into 3 mutually exclusive risk classes based on the quantiles of the predicted probability of in-hospital mortality, i.e., the lowest risk (class 1, <25% quantile), moderate risk (class 2, between 25 and 75% quantile), and highest risk (class 3, >75% quantile) for evaluation. We also calculated the internally validated Brier score to assess the overall fit of the model and internally validated Nagelkerke's *R*^2^ to measure the explained variation.

### Risk Score

To facilitate the use of the selected risk factors and the risk model, we constructed a simple risk score for each patient based on the regression coefficients estimated from the risk model with the original sample. Points for each risk factor were calculated by dividing the risk factor's coefficient by summing the absolute value of coefficients in the model, multiplying by 100, and rounding to the nearest integer. We stratified patients into three risk groups based on the distribution of the risk score: low (<25th percentile), average (25th−75th percentile), and high (>75th percentile).

Analysis was conducted using SAS statistical software version 9.4 (SAS Institute Inc.). The study followed the Transparent Reporting of a Multivariable Prediction Model for Individual Prognosis or Diagnosis (TRIPOD) reporting guideline. Each of the 22 items of the TRIPOD statement was addressed.

## Results

### Patient Characteristics

A total of 153 eligible patients were enrolled. The general characteristics of the 153 patients are shown in the Table 1. The mean age at diagnosis was 21.6 years (standard deviation 14.2) and 62 (40.5%) patients were female. Fifty-nine patients died and 94 remained continuously survival during follow-up phase. The rate of in-hospital mortality was 41.2% (95% CI, 31.6–50.7%) for the original sample. According to the outcome of patients with OS, these patients were divided into two groups named the death and survival groups.

**Table 1:** General characteristics of the 153 patients.

**Characteristics**	**Aggregate**	**Death**	**Survival**	***p-*values**
Total	153	59	94	
Age, mean age (SD) (years)	21.6 (14.2)	19.7 (12.3)	22.8 (15.2)	0.3002
Median (IQR) (years)	16 (13, 24)	16 (13, 22)	16.5 (13, 27)	
Gender, female, *n* (%)	62 (40.5)	24 (40.7)	38 (40.4)	0.9753
Tumor site, lower limbs, *n* (%)	141 (92.2)	57 (96.6)	84 (89.4)	0.1045
Tumor length of lesion, mean (SD) (cm)	10.1 (4.5)	11 (5.4)	9.5 (3.8)	0.1653
Median (IQR) (cm)	9 (6.7, 12)	10.1 (7, 13.2)	9 (6.7, 12)	
Tumor volume, mean (SD) (cm^3^)	545.6 (661.7)	710 (725.1)	442.5 (599.9)	0.0009
Median (IQR) (cm^3^)	291.2 (142.1, 680.6)	523.7 (201.1, 923.4)	250.4 (119.2, 448.5)	
Primary metastasis, *n* (%)	34 (22.2)	25 (42.4)	9 (9.6)	<0.0001
Alkaline phosphatase, mean (SD) (U/L)	246 (281.2)	297.6 (321.3)	213.6 (249.1)	0.003
Median (IQR) (U/L)	147 (104, 251)	196 (136, 346)	126 (94, 223)	
In-hospital mortality rate, % (95% CI)		41.2% (95%CI, 31.6–50.7%)		

*SD, standard deviation; IQR, interquartile range; cm, centimeter; CI, confidence interval*.

Of the extremity tumors, 141 (92.2%) were situated in the lower extremities (femur, fibula, tibia), and 12 (7.8%) were situated in the upper extremities (humerus, radius). The primary tumors involved lower limbs tumors for the death and survival groups were 57 (96.6%) patients and 84 patients (89.4%), respectively. As to tumor lesions, the median length of lesion was 10.1 centimeter [interquartile range (IQR) 7–13.2] in the death group and 9.0 centimeter (IQR 6.7–12) in the survival group. There were no significant differences in age, sex, tumor site, and tumor lesion between the two groups, while tumor volume and serum levels of alkaline phosphatase were markedly increased in OS patients with death vs. survival and primary metastasis was significantly related to the overall survival of patients (Table 1).

### Risk Factor Selection and Validation

The MCMC method selected three primitive factors with a posterior probability of at least 0.90, including lower limbs, tumor volume and primary metastasis. The final model was developed with a logistic model, and primary metastasis and tumor volume were selected.

The risk model based on the two risk factors demonstrated good discrimination, calibration, overall fit, and explained variation. The internally validated c-statistic was 0.7308. For calibration, the validated calibration slope was 0.9660. The mean observed in-hospital mortality rate ranged from 21.05% in the lowest predicted quantile to 68.42% in the highest predicted quantile, a range of 47.4%. The validated Brier score was 0.2021, and the validated explained variation was 0.2006.

### Risk Score

**Figure 3:**
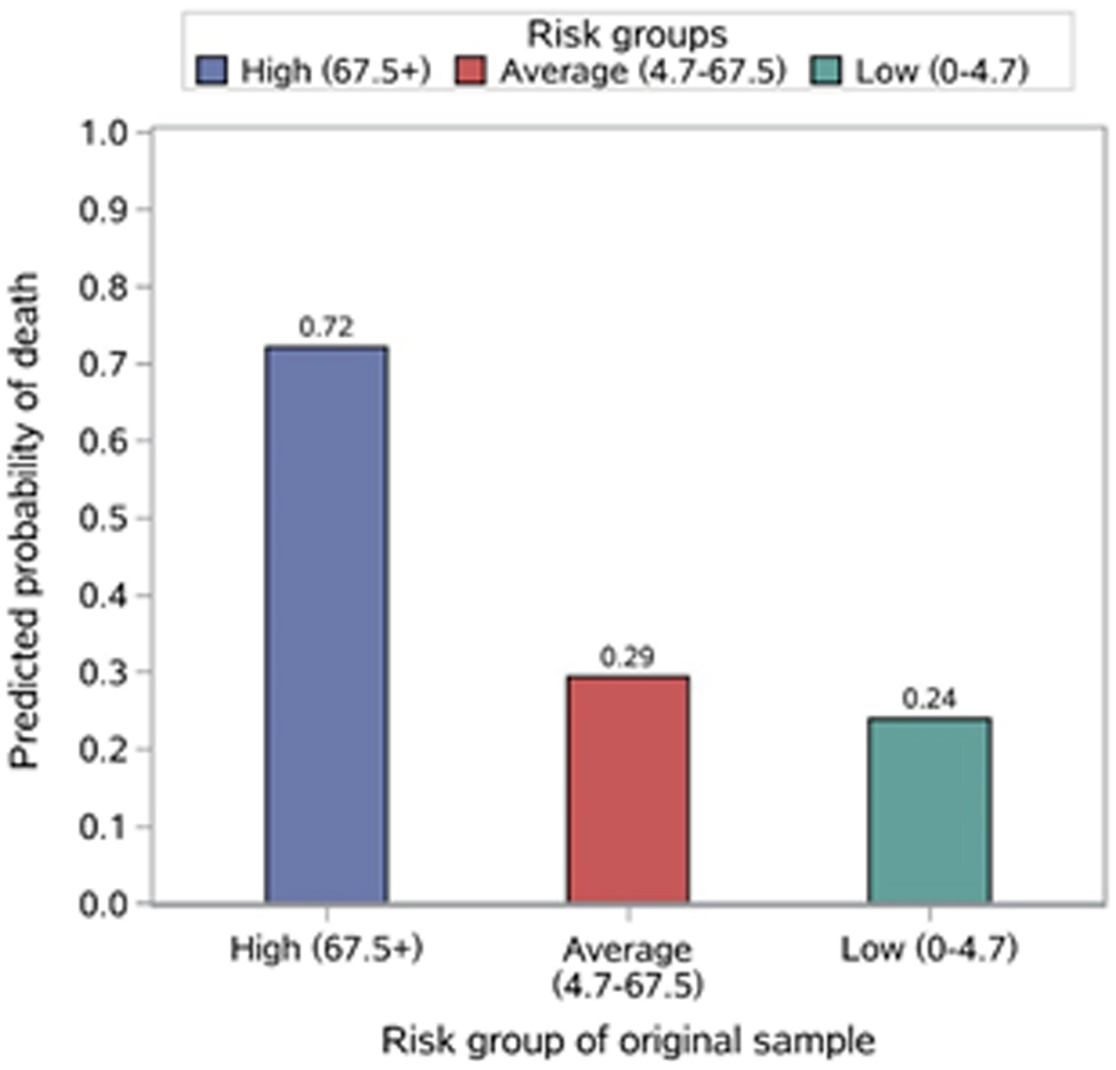
Risk score.

The risk stratification of in-hospital mortality is demonstrated in Table 2. Based on the distribution of the risk score, 24.8, 49.7, and 25.5% of patients were stratified into the high- (risk score ≥ 67.5+), average- (risk score 4.7–67.5) and low-risk (risk score 0–4.7) groups for in-hospital mortality, with corresponding probabilities of 0.684, 0.329, and 0.210, respectively (Figure 3).

**Table 2:** Patients risk stratification based on risk score.

**Risk groups**	**Patients, *n* (%)**	**In-hospital mortality, mean (%)**
High (risk score 67.5+)	38 (24.8)	68.4
Average (risk score 4.7–67.5)	76 (49.7)	32.9
Low (0–4.7)	39 (25.5)	21.0

## Discussion

Osteosarcoma is the most common primary malignant bone tumor with higher rates of invasion and metastasis. Although these therapeutic regimens for osteosarcoma have been unprecedentedly advanced, the prognosis is still poor in patients with OS (17). The 5-year overall survival rate of OS patients has remained virtually unchanged in recent decades, especially for metastatic osteosarcomas (6, 17). This may be due to the rarity and heterogeneity of the tumor, together with the lack of pathognomonic mutations identified and the limited targeted treatments thus far (3, 7, 17, 18). Thus, a novel and innovative risk model predicting mortality is urgently needed to increase the understanding of factors identified to exert prognostic effects in patients with OS.

Several clinical trials have been performed to identify the prognostic indicators relevant to the mortality of OS patients, which have been mostly conducted in developed countries. However, the outcomes vary among these studies. The common clinically detected tumor size is widely evaluated in prognostic modeling, whereas the predictive performance of tumor size in predicting outcome was inconsistent among different studies. Some studies indicated that tumor size was one of the significant prognostic factors to predict in-hospital mortality in OS patients (9, 19–22), while tumor size lost its significance in another study; it did not appear to be a significant prognostic factor at all (13). Moreover, worldwide, there is still no consensus as to the standardized risk model that can be used to evaluate the prognosis of OS. Thus, in the absence of availability of these clinical data (such as histologic response to chemotherapy and type of surgery and surgical margins) before the treatment of OS cases, it is difficult to determine whether the prognostic factor evaluated is the true prognostic effect or not and to apply the proposed risk factors to stratify patients at diagnosis before treatment.

We conducted this study based on the patient cohort with OS. First, we analyzed the clinical data of OS cases and selected these potential prognostic variables associated with the outcome. These clinical data, as predictive factors, are conveniently collected, widely reliable, and clinically important during hospitalization and could be applied to predict the outcomes of OS cases by statistical algorithms. Then, we employed MCMC simulation to explore the strength of the correlation between these factors and the prognosis of patients with OS. Two risk factors (metastasis, tumor volume) were identified with the aggressive regimens, which were independent predictive indicators of 5-year survival in OS cases. Additionally, their prognostic value has been recognized in many studies (8–10, 23, 24). Finally, a simple risk model was constructed and evaluated based on the two factors, which reflected good discrimination, calibration, overall fit, and explained variation. We further constructed a simple risk score to stratify patients into three risk groups of in-hospital mortality. Through the risk stratification, we found that 24.8% of the patients were at high risk of in-hospital mortality, which emphasized the importance of identifying these patients to provide them with targeted and systemic treatment and establish appropriate surveillance strategies. Moreover, on the foundation of the risk model, we are able to offer OS cases useful prognostic information and predict survival at diagnosis. Thus, the results of this study demonstrated that not only this risk model but also the risk score had an important potential application in clinical work.

Despite the advantages outlined above, our study has several limitations. First, the study did not have sufficient OS patients at new diagnosis. The main reason is the rarity of the disease and the difficulty in accumulating adequate cases. Second, this was a single-center retrospective study in China, and the performance of the risk model lacked validation in more independent regions and different races. Third, our results were based on the foundation of the existing medical records database and lacked the independent and external validity of this study. Finally, further studies are needed to estimate and confirm the generality of our results.

## Conclusion

In summary, in this study, we established a novel prognostic risk model based on clinical data from OS patients at primary diagnosis in China. It may help clinicians stratify patients into different risk groups of in-hospital mortality, provide them with targeted and systemic treatment and establish appropriate surveillance strategies. Hence, these findings offer a direction to predict the prognosis of OS cases.

## Data Availability Statement

The original contributions presented in the study are included in the article/supplementary material, further inquiries can be directed to the corresponding author/s.

## Ethics Statement

The studies involving human participants were reviewed and approved by the Ethics Committee of Affiliated Cancer Hospital of Zhengzhou University. Written informed consent for participation was not provided by the participants' legal guardians/next of kin because: The clinical study was retrospective involving human participants. Only the clinical data (gender, age, tumor site, etc.) of hospitalized patients with osteosarcoma are collected, it does not involve intervention in the treatment and prognosis of patients, and does not bring risks to the physiology of patients. Meanwhile, we will do our best to protect patient information and not disclose patient privacy. The Ethics Committee waived the requirement for written informed consent for participation.

## Author Contributions

YC and ZR designed the study. YC, CL, XW, and ZL retrieved references and analyzed data. YC and CL wrote the manuscript. ZR revised the manuscript. All authors reviewed and approved the manuscript.

## Funding

This study was sponsored by grants from National Key Research and Development Program of China (2018YFC2000501), National Natural Science Foundation of China (U2004121), and China Postdoctoral Science Foundation (2020T130609).

## Conflict of Interest

The authors declare that the research was conducted in the absence of any commercial or financial relationships that could be construed as a potential conflict of interest.

## Publisher's Note

All claims expressed in this article are solely those of the authors and do not necessarily represent those of their affiliated organizations, or those of the publisher, the editors and the reviewers. Any product that may be evaluated in this article, or claim that may be made by its manufacturer, is not guaranteed or endorsed by the publisher.
